# Is it necessary to approach the Heavy-work investment concept in ophthalmology services? The double valence of workaholism

**DOI:** 10.22336/rjo.2020.53

**Published:** 2020

**Authors:** Mădălina Gheorghe Consuela

**Affiliations:** * Philologist, Authorized translator

The pandemic context has changed many perspectives during 2020 and managers or CEOs had to find other efficient strategies for sustainable work environment. However, in health care services, and implicitly in ophthalmology services, the challenge became more prominent due to the staff’s involvement and core importance. Since health care services are intangible and are the most personalized types, the staff was forced to satisfy the needs and the wants of consumers but with some mental and physical pressure due to the high rate of SARS-CoV-2 infection. As such, the meaning of work has attracted a great deal of debate and received a lot of attention because of the dynamic changes in the work environment and patterns. 

Heavy-work investment was first introduced in scientific literature in 2012 by Snir and Harpaz, in the article entitled “Beyond workaholism. Towards a general model of heavy-work investment”, published in Human Resource Management Review. In their opinion, heavy-work investment has been elaborated on the principle that people hold different ideas about the value and the consequences of working hard. Accordingly, there is a negative heavy-work investment subtype that depends on the context or situation, called workaholism and a positive heavy-work investment subtype, which depends on the disposition, namely work engagement.

Moreover, Snir R and Harpaz I also mention that there are several heavy-work investor typologies that are worth mentioning, with a high appliance in ophthalmology services as well. Thus, in the situational category, the heavy-work investors may be the needy, characterized by individuals who support large families, pay debts, and the employer-directed category that encompasses high-tech workers and hospital physicians. The two categories have in common the fact that the urge to work is on short term, uncontrollable and stable. In contrast, the dispositional category refers to the following heavy-work investors:

- The workaholics are the individuals who are addicted to their work and their main characteristic is their internal, uncontrollable and stable urge to work;

- The work-devoted are persons who exhibit a high passion for their work, which comes from inside, is controllable and is also stable;

- The intimacy-avoiders are the individuals who consider work as an escape from close relationships. Their main characteristic resides in the fact that their urge to work is internal, controllable and stable;

- The materialists are persons who strive for a high standard of living and their main particularity is that their urge to work comes from inside, is controllable as well as stable.

Taking into account the above-mentioned categories, it can be concluded that, in ophthalmology services and due to the pandemic context, workaholism can have a double valence, that is, dedication, the positive valence and the workaholism per se, due to organizational and external forces. 

Actually, workaholism is defined by the fact that some employees spend more time, energy and resources to fulfill their tasks at work than others. However, this trait seems to be related to each individual’s personality. When referring to the term “workaholic”, we may refer to people whose need to work has become so exaggerated that it may constitute a danger to their health, personal happiness, interpersonal relations and social functioning. As it can be seen, it is regarded more as a negative term than a positive one, or at least negative for the individual who is a workaholic and positive for the organization that has such an employee and also for the benefit of the community. Consequently, workaholism is a positive trait of an employee, as it involves a pleasurable engagement at work. This suggests that the individual who is regarded as a workaholic does nothing wrong, but is happy and content that he is given the opportunity to do what he likes for as long as his physical and mental condition allows him to do it. 

Nowadays, organizations tend to hire such dedicated individuals, who are in fact workaholics, this way encouraging the process of heavy-work investment. Thus, workaholism is somehow triggered by the work enjoyment and the job satisfaction. This means that ophthalmological organizations have to reward the workaholic employees but ensure a healthy work environment, so as to become a Learning Organization as mentioned by Gheorghe CM, Purcărea VL, Gheorghe IR, Popa-Velea O in the article entitled “Investigating the dimensions of learning organizations questionnaire (DLOQ) in a Romanian private ophthalmology organization” and published in 2019 in Romanian Journal of Ophthalmology. 

